# Mueller matrix polarimetry for quantitative evaluation of the Achilles tendon injury recovery

**DOI:** 10.1007/s12200-024-00142-2

**Published:** 2024-12-09

**Authors:** Huibin Yang, Minhui Xu, Honghui He, Nan Zeng, Jiawei Song, Tongyu Huang, Ziyang Liang, Hui Ma

**Affiliations:** 1 Shenzhen International Graduate School, Tsinghua University, Shenzhen, 518055 China; 2 School of Teacher Education, Nanjing Normal University, Nanjing, 210097 China; 3 Department of Spinal Orthopedics and Massotherapy in Chinese Medicine, Fourth Clinical Medical College of Guangzhou University of Traditional Chinese Medicine, Shenzhen, 518022 China; 4 Department of Physics, Tsinghua University, Beijing, 100084 China

**Keywords:** Mueller matrix, Achilles tendon injury, Polarimetry

## Abstract

Achilles tendon injuries, as a widely existing disease, have attracted a lot of research interest. Mueller matrix polarimetry, as a novel label-free quantitative imaging method, has been widely used in various applications of lesion identification and pathological diagnosis. However, focusing on the recovery process of Achilles tendon injuries, current optical imaging methods have not yet achieved the label-free precise identification and quantitative evaluation. In this study, using Mueller matrix polarimetry, various Achilles tendon injury samples were characterized specifically, and the efficacy of different recovery schemes was evaluated accordingly. Experiments indicate that injured Achilles tendons show less phase retardance, larger diattenuation, and relatively disordered orientation. The combination of experiments with Monte Carlo simulation results illustrate the microscopic mechanism of the Achilles tendon recovery process from three aspects, that is, the increased fiber diameter, a more consistent fiber orientation, and greater birefringence induced by more collagen protein. Finally, based on the statistical distribution of polarization measurements, a polarization specific characterization parameter was extracted to construct a label-free image, which cannot only intuitively show the injury and recovery of Achilles tendon samples, but also give a quantitative evaluation of the treatment.

**Graphical Abstract:**

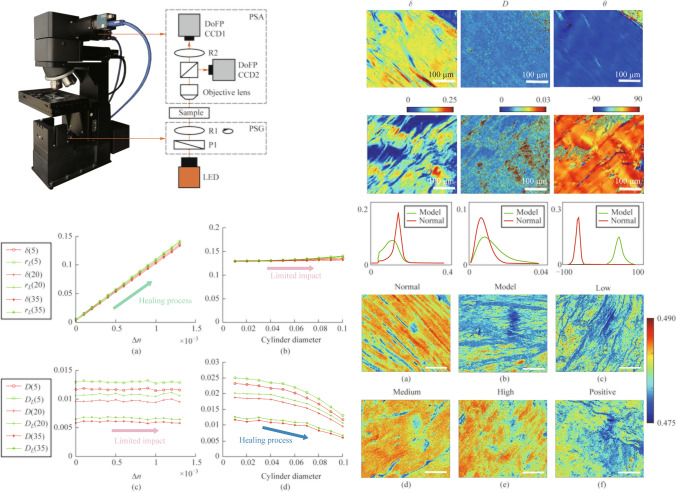

## Introduction

Tendons are composed of complex fibrous connective tissue containing extracellular matrix (ECM) and cells. The stretching ability of tendons is mainly determined by ECM, and collagen fibers account for 60% to 85% of the dry weight of ECM [1, 2]. The anisotropy of tendons originates from neatly arranged collagen fibers [3], so changes in fibrous biological structures are the most prominent microphysical characteristics associated with anisotropy of tendon injury and lesions.

The recovery process after natural tendon injuries consists of three overlapped stages, the inflammatory stage, the proliferative stage, and the remodeling stage [4]. The inflammatory stage involves inflammatory cell infiltration, synthesis of ECM components, and angiogenesis stimulated by the inflammatory response. In the proliferative stage, large numbers of cells are produced and ECM components are deposited in a randomized manner. In the remodeling stage, the density of cells reduces, the arrangement of collagen fibers in the ECM becomes aligned, and the proportion of internal components changes. There is no clear boundary between these three stages, and the full complexity of the natural healing process remains incompletely elucidated at present.

The Achilles tendon is one of the most commonly injured parts of the human body, and its injury is a common orthopedic traumatic disease in clinical practice [1, 5]. Due to its limited natural regenerative capacity, many methods to promote the recovery of the Achilles tendon have been proposed. Except for most notably conservative treatments and surgical treatments, some emerging methods such as tissue engineering have also been proposed [6]. With the increasing number of emerging treatment options, corresponding scientific evaluation methods and tracking interpretation techniques are also key issues in tendon research.

For imaging methods used for the examination of Achilles tendon injuries, MRI and ultrasound are commonly used in vivo methods in the clinic as diagnostic supports for physicians [7], but only non-quantitative results can be obtained when we evaluate the treatment effects. Notably, significant changes in collagen fibers during Achilles tendon recovery have motivated the exploration of novel imaging techniques, including second-harmonic generation (SHG) microscopy [8], polarization-sensitive optical coherence tomography (PS-OCT) [9], polarized light microscopy [10], and so on. However, these methods exhibit limitations such as low imaging resolution, limited characterizations, and the need for staining.

In the last few years, Mueller matrix (MM) polarimetry has emerged as a valuable tool for label-free biomedical imaging applications. It holds the potential for non-invasive quantitative detection of pathological tissues, such as fibrosis, and has become an important metric in the diagnostic processes of various pathological conditions. For instance, different stages of ductal carcinoma in the breast exhibit typical differences in the proportion of fibrous tissue and the pattern of fibrous arrangement outside the ducts [11, 12]. Similarly, Crohn’s disease and intestinal tuberculosis, two inflammatory bowel diseases with very similar clinical manifestations, can be distinguished by the different distributions of fibrosis around granulomas [13]. Moreover, MM polarimetry has shown potential in differentiating various types of fibers in tissues, such as collagen fibers and muscle fibers [14, 15]. This matrix polarized imaging mode can be connected with richer tissue microscopic features than non-polarization images, and on the other hand, it also poses challenges for specific biological information extraction. In previous studies, the extraction and validation of specific polarization parameters usually originated from clear physical deductions or supported by reasonable tissue optical Monte Carlo simulations. Furthermore, the introduction of emerging data processing algorithms has also provided a new analytical approach for the fusion of high-dimensional and multi-parameter optical experimental indicators to characterize a specific type of tissue lesion or damage. Dong et al. employed the gray level co-occurrence matrix (GLCM) to achieve quantitative characterization of changes in breast cancerous tissues at different stages and regions [16]. Yao et al. proposed a radiomics method based on polarization imaging, providing quantitative diagnostic features for staging liver fibrosis and indicating the microstructure changes [17].

While the majority of MM polarimetry applications have been confined to 2D imaging, the exploration of 3D polarization imaging presents an exciting frontier. For instance, research has extended the concept of MM imaging to the domain of 3D integral imaging, enabling the estimation of polarization degree for any arbitrary composite light source on any selected plane [18]. Furthermore, studies have introduced polarization holographic Mueller matrix methods for assessing three-dimensional morphology, with applications in cancer detection and diagnosis [19, 20]. Additionally, investigations into 3D Mueller matrix mapping methods have analyzed the spatial variations of Mueller matrix invariants in histological sections [21]. In another advancement, the functionality of second-harmonic polarization techniques has been expanded to determine the three-dimensional geometry of collagenous tissues, offering deep insights into tissue structure without the need for multiple scans or intricate post-processing [22]. However, due to the complexity of the principles behind 3D polarimetry, the parameter system is not yet fully enriched, and there is a lack of validation in actual clinical scenarios, it still requires further development.

Polarization measurements can be a suitable way to track the microstructural changes during the recovery from Achilles tendon injuries, as well as modifications in collagen organization. In this paper, we measured normal and injured Achilles tendon samples using MM microscopy. Considering the observed strong anisotropy, the study extracted and analyzed three types of polarization basic parameters (PBPs), including retardance, diattenuation, and angular parameters, and then evaluated the recovery effects of injured Achilles tendon samples in different treatment groups. Next, we explained the recovery mechanism of injured Achilles tendon samples under different treatment levels with the help of Monte Carlo calculations including tendon tissue modeling and polarization imaging simulation. Finally, based on the differences in PBPs’ distribution between the normal and injured samples, a polarization feature parameter (PFP) describing the recovery of Achilles tendon injuries is extracted. Compared with the single PBP, the proposed PFP can effectively enhance the inter-class differences among the two different groups of tendons, that is, enhance the contrast of polarization characterization of tendon injuries. The PFP can be a basis for evaluating various therapeutic regimens from the perspective of polarization optics, and the polarization virtual staining also provides an unmarked image to intuitively highlight the key areas in tendon injury images.

## Methods and materials

### Sample preparation

SPF-grade SD male rats aged 10–12 weeks were used to establish a disease model through conventional Achilles tendon injury modeling methods. Three days before surgery, botulinum toxin (Botox, Allergan, Irvine, CA) was injected into the gastrocnemius muscle of both hind limbs of the rats to induce plantar flexor paralysis (3U/rat, total volume of 0.06 mL), which reduced the passive mechanical load on the muscles after Achilles tendon injury. The fur on the hindlimbs was shaved one day before the surgery, followed by complete depilation using depilatory cream. During the surgery, the rats were fixed in a supine position with a warming pad to maintain their body temperature, and anesthesia was administered through intraperitoneal injection of pentobarbital sodium (50 mg/kg) in rats weighing about 250 g. The depilated skin of the rat’s hind limb was thoroughly disinfected with iodophor, and a transverse incision was made on the lateral side of the hind limb Achilles tendon to expose the Achilles tendon complex. The Achilles tendon was transected by 3/4, followed by skin suturing and disinfection. The experiment was divided into six groups: ① Normal group; ② Model group; ③ Shallow stimulation group; ④ Moderate stimulation group; ⑤ Deep stimulation group; ⑥ Positive group. Group ① consists of healthy, normal rat tissue samples, as a reference. The model group ② is derived from transversely sectioned Achilles tendon samples. Subsequently, groups ③–⑥ used an adjustable hindlimb brace to prevent joint movement after Achilles tendon injury. The brace was composed of two parts fixed by four screws, made of 3D-printed resin material, weighing about 15 g. The rat’s ankle was fixed in a plantarflexed position, with the upper edge of the fixation spanning the knee joint and the lower edge reaching the upper one-third of the plantar surface. In the positive group, the fixation brace was removed two weeks after the conventional Achilles tendon injury, and the rats were allowed to move freely. After one week of fixation, the shallow, moderate, and deep stimulation groups received mechanical force stimulation, where an in-house developed external mechanical device applied slow downward pressure on the superficial, middle, and deep layers of the muscle, respectively. Simultaneously, the overall tissue deformation of the stimulated limb was visually observed. A stainless-steel bracket was placed under the rat, and fixed with ropes when necessary. The three groups were randomly assigned to receive 10 min of mechanical pressure stimulation on the posterior triceps calf region of the rat’s hindlimb every 2 days, with a treatment duration of two weeks. The groups ③–⑥ were subjected to various treatment regimens collectively referred to as the treatment groups, and their therapeutic effects need to be further analyzed. We prepared unstained, deparaffinized pathological sections, each 10 μm thick, from the samples of all groups for Mueller matrix microscopy.

### MM microscopy

**Fig. 1:**
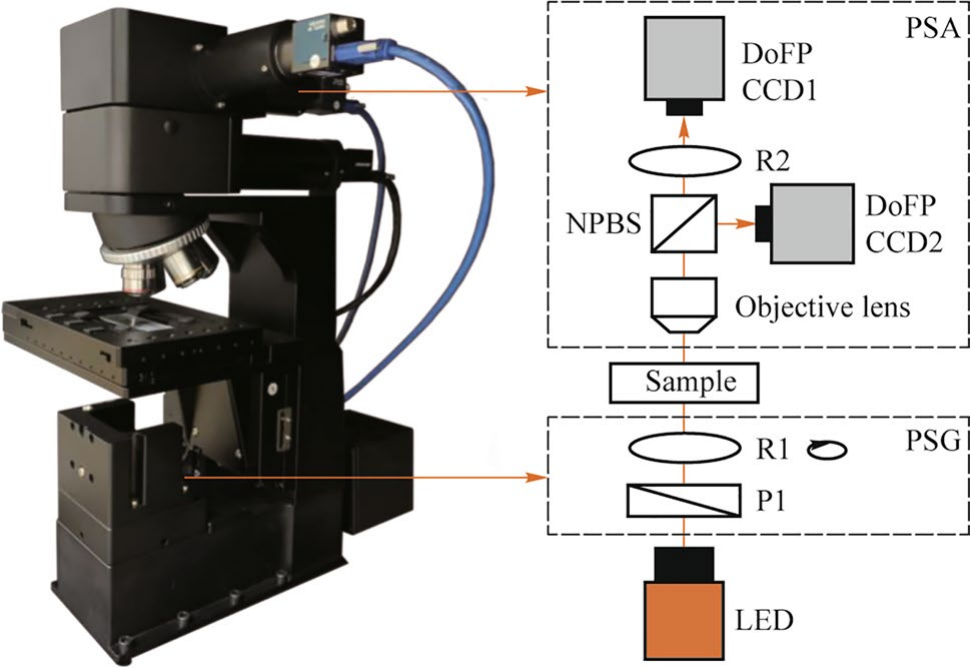
Schematic of the MM microscopy based on the dual division of focal plane (DoFP) polarimeters

We obtain the MM images of the pathological samples by the upright transmission MM microscopy based on the dual division of focal plane (DoFP) polarimeters shown in Fig. 1. The system adopts the upright transmission optical geometry. The illuminating light from the LED (633 nm, Δ*λ* = 20 nm) is collimated by a lens and modulated by a polarization state generator (PSG). After interacting with the sample, the transmitted light passes through the objective lens (20 × /0.4 NA) and is finally detected by a polarization state analyzer (PSA). The PSG module is equipped with a fixed-linear polarizer (P1, FLP13-VIS-M, Lubon Optics, China) and a rotating zero-order quarter-wave plate (R1, QWP10-633B, Lubon Optics, China), with R1 being mounted on an automated rotational platform. The PSA is assembled with a 50:50 beam splitter prism (NPBS, CCM1-BS013/M, Thorlabs Inc), a quarter-wave plate set at a constant angle (R2, QWP25-633A, Lubon Optics, China), and two DoFP cameras (MER2-503-23GM-P, Daheng Optics). Within the PSA, the DoFP-CCD1 and DoFP-CCD2 cameras, which share the same field of view (FOV), resolution, and exposure duration, are fixed to two ends of the prism and are oriented to align with the 0° polarization axis. The pixelated micro-polarizer array enables the DoFP polarimeter to capture images representing four distinct linear polarization states: 90°, 45°, 135°, and 0°, all in a single snapshot. In the PSG, a static linear polarizer P1 and a variable-angle quarter-wave plate R1 are utilized to alter the polarization state of the incoming light. The alignment of P1’s polarization and R1’s initial fast axis is set to 0°, matching the 0° polarization direction of the two DoFP polarimeters. Once the PSG has produced at least four independent polarization states $[S_{{{\text{in}}}} ]$, the PSA yields the polarization state to be measured $[S_{{{\text{out}}}} ]$, thereby enabling the acquisition of MM images $M = [S_{{{\text{out}}}} ][S_{{{\text{in}}}} ]^{ - 1}$ [23].

### Polarimetry basis parameters

Although MM contains a large amount of information that reflects the microstructural characteristics of the tested sample, a single matrix element does not have a clear and independent physical representation ability. Concerned with this issue, a series of polarization parameter extraction methods have been proposed, such as Mueller matrix polarization decomposition (MMPD) [24], and Mueller matrix transformation (MMT) parameters [25], whose physical meanings have been investigated in previous studies. Tables 1 and 2 list the PBPs involved in Achilles tendon-related research, and the corresponding physical meanings are also provided based on previous research [20].

**Table 1:** PBPs of MMPD

Polarimetry basis parameters (PBPs) of MMPD	Physical meaning
$D={m}_{11}^{-1}\sqrt{{m}_{12}^{2}+{m}_{13}^{2}+{m}_{14}^{2}}$	Diattenuation
$\Delta =1-\frac{1}{3}|\text{tr}{M}_{\Delta }|\in [\text{0,1}]$	Degree of overall depolarization
$\delta =\text{arccos}\left(\sqrt{{\left({M}_{R22}+{M}_{R33}\right)}^{2}+{\left({M}_{R32}-{M}_{R23}\right)}^{2}}-1\right)$	Linear retardation
$\alpha =\text{arctan}\left(\left({M}_{R32}-{M}_{R23}\right)/\left({M}_{R22}+{M}_{R33}\right)\right)$	Angle of optical rotation
$\theta =\frac{1}{2}\text{arctan}({M}_{R42}-{M}_{R24}/{M}_{R34}-{M}_{R43})$	Fast axis orientation angle

**Table 2:** PBPs of MMT

Polarimetry basis parameters (PBPs) of MMT	Physical meaning
${m}_{14},{m}_{41}$	Associated with circular diattenuation effect
${\text{CD}}={m}_{14}+{m}_{41}$	Degree of circular dichroism anisotropy
${m}_{44}$	Related to circular depolarization
${D}_{L}=\sqrt{{m}_{12}^{2}+{m}_{13}^{2}}\in \left[0,1\right]$	Linear diattenuation
${P}_{L}=\sqrt{{m}_{21}^{2}+{m}_{31}^{2}}\in \left[0,1\right]$	Linear polarizance
$$\begin{array}{c}{q}_{L}=\sqrt{{m}_{42}^{2}+{m}_{43}^{2}}\in \left[0,1\right]\\ {r}_{L}=\sqrt{{m}_{24}^{2}+{m}_{34}^{2}}\in \left[0,1\right]\end{array}$$	Capability of transforming between linear and circular polarizations
${t}_{1}=\frac{1}{2}\sqrt{{\left({m}_{22}-{m}_{33}\right)}^{2}+{\left({m}_{22}+{m}_{33}\right)}^{2}}$	Degree of overall linear anisotropy
$A=\frac{2b{t}_{1}}{{b}^{2}+{t}_{1}^{2}}$	Degree of overall linear anisotropy
$|\mathbf{B}|={m}_{22}{m}_{33}-{m}_{23}{m}_{32}$	Determinant of MM central block
$\| \mathbf{B}\| =\sqrt{{m}_{22}^{2}+{m}_{23}^{2}+{m}_{32}^{2}+{m}_{33}^{2}}$	Frobenius norm of MM central block
${\alpha }_{r}=\frac{1}{2}{\text{arctan}}2(-{m}_{24},{m}_{34}$)	Orientation parameter corresponding to linear retardance

### Evaluation system based on PBPs of Achilles tendon recovery

In previous studies, MM images, as well as PBPs, have been frequently used for direct intuitive comparative analysis. To further quantify the differences, the frequency distribution curves and their first-order statistics can be analyzed. As we mentioned in the previous section, there are rotation-invariant and rotation-variant parameters in the PBPs. For rotation-variant parameters, it is meaningless to compute the mean value, so we compute the standard deviation of rotation-invariant parameters. For rotation-invariant parameters, we take the mean value as the main statistic.

Using normal healthy tissue samples as a reference, the therapeutic efficacy can be objectively evaluated by comparing the first-order statistics of the PBPs from normal groups and other groups. Assuming the statistical value of a certain PBP for normal group samples is *p*_n_. For rotation-invariant parameters, *p*_n_ stands for the mean value *m*_n_. For rotation-variant parameters, *p*_n_ stands for the standard deviation *δ*_n_, the quantitative evaluation indicator of each sample is defined as Eq. (1),

1 $$S_{{{\text{parameter}}}} = \frac{p}{{p_{{\text{n}}} }},$$

where *p* is the statistical value of the polarization parameter for the sample to be evaluated.

### Monte Carlo simulation for interpreting injured Achilles tendon recovery mechanism

In previous studies, a Monte Carlo program combined with a sphere-cylinder birefringence tissue model (SCBM) [26] has been successfully used to explain the polarization contrast in tissue imaging. In simulations, complex biological tissues can be modeled as a combination of spherical scatterers, cylinder scatterers, and ambient medium with birefringence, and the transmitted and scattered photon behaviors and their polarization states in tissues are simulated and recorded. For Achilles tendon samples, cylinder scatterers symbolize the collagen fibers, and sphere scatterers symbolize the nuclei and organelles. Variables that can be adjusted include the scatterer size, scattering coefficient, refractive index, and the orientation distribution of fibrous structure, as well as the birefringence and refractive index of the surrounding medium. By adjusting the model settings accordingly based on pathological phenomena, various microstructural changes of the measured Achilles tendon samples under different treatment levels can be simulated.

### PFP parameters based on polarization distribution differences

In the previous section, we computed the frequency distribution histogram (FDH) for a series of PBPs and extracted first-order statistics such as mean and standard deviation for the comparisons between the normal group and the injured group. However, the high-dimensional indicator system constructed by all PBPs is too cumbersome and not suitable for specific pathological phenomenon characterization.

**Fig. 2:**
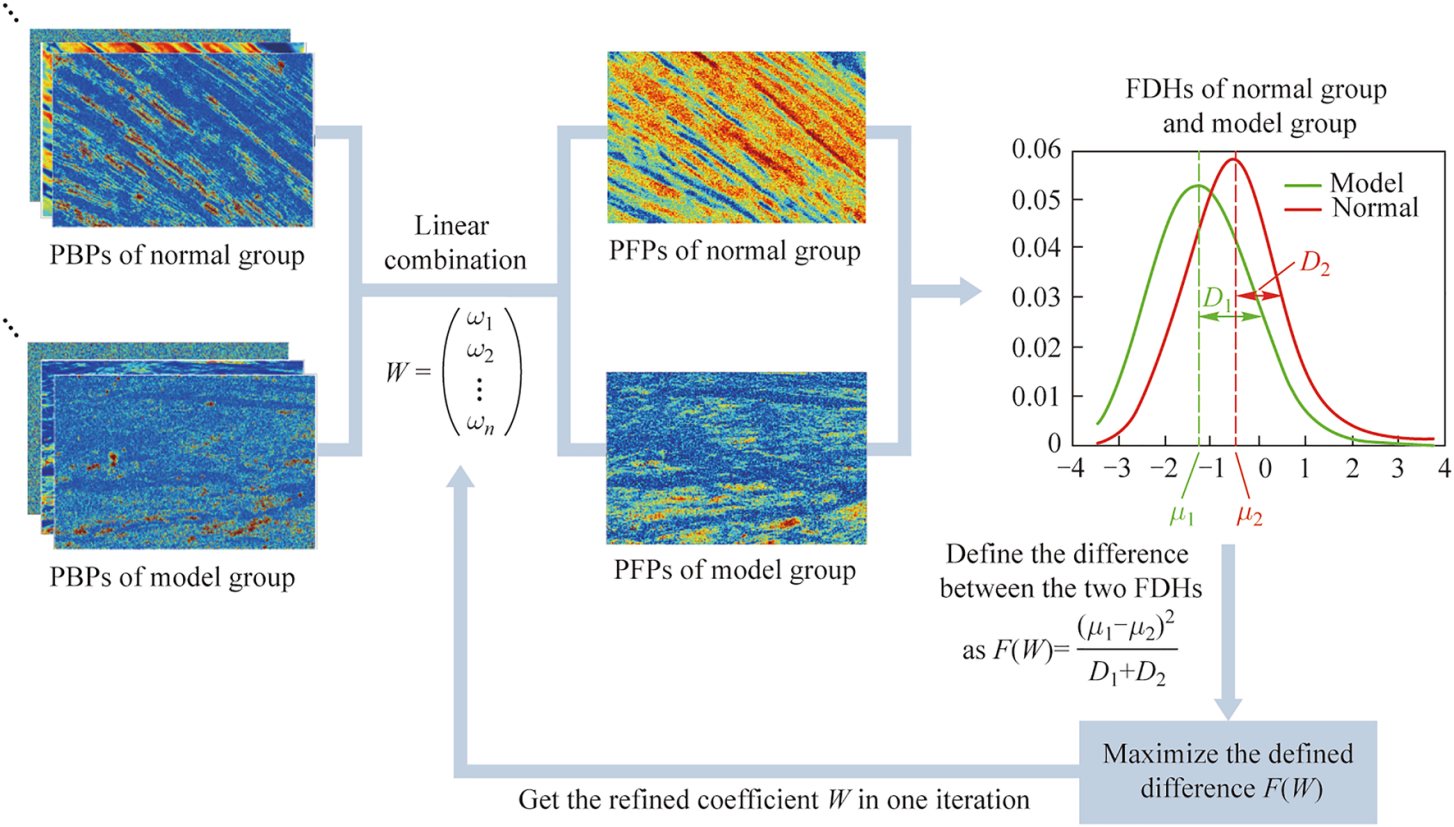
Schematic for generating PFP based on distribution differences

To enhance the specificity of the polarization index in characterizing tendon injury, we need to extract more effective PFP indicators from PBP indicators. The extraction method by linearly weighting the PBPs allows us to see the proportion of various PBPs in the PFP, which is more interpretable compared to other methods such as deep learning [27, 28]. These weighting coefficients are selected to maximize the polarization distribution differences between groups of different tendon states, and the schematic of our algorithm is shown in Fig. 2.

We define the PBP vector for each pixel point in the sample as *x*_i_ with the dimension of n*1, *n* is the number of selected PBPs. The weighting coefficients are denoted as $W = \left( {\omega_{1} ,\omega_{2} ,...,\omega_{n} } \right)^{{\text{T}}}$, so every pixel in PFP is denoted as $y_{i} = W^{{\text{T}}} x_{i}$. To maximize the difference between the probability distribution of PFP for the normal sample $P{(}y_{i} \left| {x_{i} \in C_{1} } \right.{)}$ and the injured model sample $P{(}y_{i} \left| {x_{i} \in C_{2} } \right.{)}$, $P{(} \cdot {)}$ is used to define the probability distribution of the random variable, *C*_1_ denotes the pixel set from the normal sample group, and *C*_2_ denotes the pixel set from the injured model group. Here we define the distribution differences as Eq. (2),

2 $$F\left( W \right) = \frac{{\left( {\tilde{\mu }_{1} - \tilde{\mu }_{2} } \right)^{2} }}{{D_{1} + D_{2} }},$$

where $\tilde{\mu }_{1}$ and $\tilde{\mu }_{2}$ are the projections centers of PFP for the two groups, respectively, on *W*. $\tilde{\mu }_{j} = W^{{\text{T}}} \mu_{j}$, $\mu_{j} = \frac{1}{{N_{j} }}\sum\limits_{{x_{i} \in C_{j} }} {x_{i} }$, $D_{j} = \frac{1}{{N_{j} }}\sum\limits_{{x_{i} \in C_{j} }} {(W^{{\text{T}}} x_{i} - W^{{\text{T}}} u_{j} } )^{2}$, *N*_*j*_ denotes the number of sample pixels in the two groups,$j \in [1,2]$.

The maximized *F*(*W*) means the maximized difference between the PFP means of the two groups $\left( {\tilde{\mu }_{1} - \tilde{\mu }_{2} } \right)^{2}$ and the minimized sum of distribution widths of the two groups *D*_1_ + *D*_2_. Further, we can analyze *F*(*W*), we can present *F*(*W*) as Eq. (3),

3 $$F\left( W \right) = \frac{{W^{{\text{T}}} S_{u} W^{{\text{T}}} }}{{W^{{\text{T}}} S_{d} W^{{\text{T}}} }},$$

where $$S_{u} = \left( {\mu_{1} - \mu_{2} } \right)\left( {\mu_{1} - \mu_{2} } \right)^{{\text{T}}}$$, $$S_{p} = \sum\limits_{{x_{i} \in C_{1} }} {\left( {x_{i} - \mu_{1} } \right)\left( {x_{i} - \mu_{1} } \right)^{{\text{T}}} } + \sum\limits_{{x_{i} \in C_{2} }} {\left( {x_{i} - \mu_{2} } \right)\left( {x_{i} - \mu_{2} } \right)^{{\text{T}}} }$$.

Either *S*_*u*_ or *S*_*p*_ is a certain value determined by the PBPs of samples. In contrast to previous approaches reliant on classifier training, we find the optimum value of *W* by calculating the extreme value of *F*(*W*). When $\frac{\partial F(W)}{{\partial W}} = 0$, the extreme value is obtained, meaning $S_{u} W = \lambda S_{p} W$ and $\lambda = F(W) = \frac{{W^{{\text{T}}} S_{u} W^{{\text{T}}} }}{{W^{{\text{T}}} S_{d} W^{{\text{T}}} }}$. Since *λ* is a number and not a matrix, it is possible to multiply both sides of the equation by $S_{p}^{ - 1}$ together, with $S_{p}^{ - 1}$ denoting the pseudo-inverse of the matrix $S_{p}$. At this point, we get

4 $$S_{p}^{ - 1} S_{u} W = \lambda W.$$

From Eq. (4), to maximize *λ*, i.e., *F*(*W*), we need to find the largest eigenvalue of $S_{p}^{ - 1} S_{u}$, and the corresponding projection direction is the optimal weighting coefficient *W*, which can be realized by using the singular value decomposition (SVD) [29].

In summary, as shown in Fig. 2, we constructed the PFP based on the combination of linearly weighted PBPs and set the function describing the PFP distribution difference from the two types of samples, and finally, with the help of the SVD, we obtained the optimal linear weighting coefficients in a single iterative process.

### Quantitative assessment of Achilles tendon injury based on PFP

The PFP obtained in the previous section can maximize the distribution differences between the normal and model groups, and since our PFP is a pixel-level superposition, there are no problems with resolution degradation similar to those methods by calculating texture features. Compared with the original light-intensity image, PFP images with the same resolution can intuitively and qualitatively display and assess the recovery effect of Achilles tendon injuries. In addition, to further quantitatively characterize the PFP distribution differences among several treatment groups and the normal group as well as the model group, we defined an evaluating indicator for treatment effects as follows:

5 $$S = 50\left( {\left( {\frac{{\left| {\tilde{u}_{r} - \tilde{u}_{2} } \right| - \left| {\tilde{u}_{r} - \tilde{u}_{1} } \right|}}{{\left| {\tilde{u}_{r} - \tilde{u}_{2} } \right| + \left| {\tilde{u}_{r} - \tilde{u}_{1} } \right|}}} \right) + \frac{{D_{1} }}{{D_{r} }}} \right),$$

where $\tilde{u}_{r}$ and *D*_*r*_ denote the mean value and the variance for the treatment groups, respectively.

The initial term (ignoring the coefficient of 50) in Eq. (5) serves to illustrate the difference between the mean PFP value of samples within the treatment group and those of both the normal and model samples. The higher first term of *S* means that the mean PFP value of the treatment group is closer to that of the normal group and more different from the model group, implying a favorable recovery effect. Subsequently, the second term characterizes the relative magnitude of the PFP distribution width within the experimental group. A narrower distribution width for the treatment group signifies an enhanced sample consistency, thereby indicating a better recovery effect.

## Results

### Comparison of normal and injured model Achilles tendon samples on PBPs

**Fig. 3:**
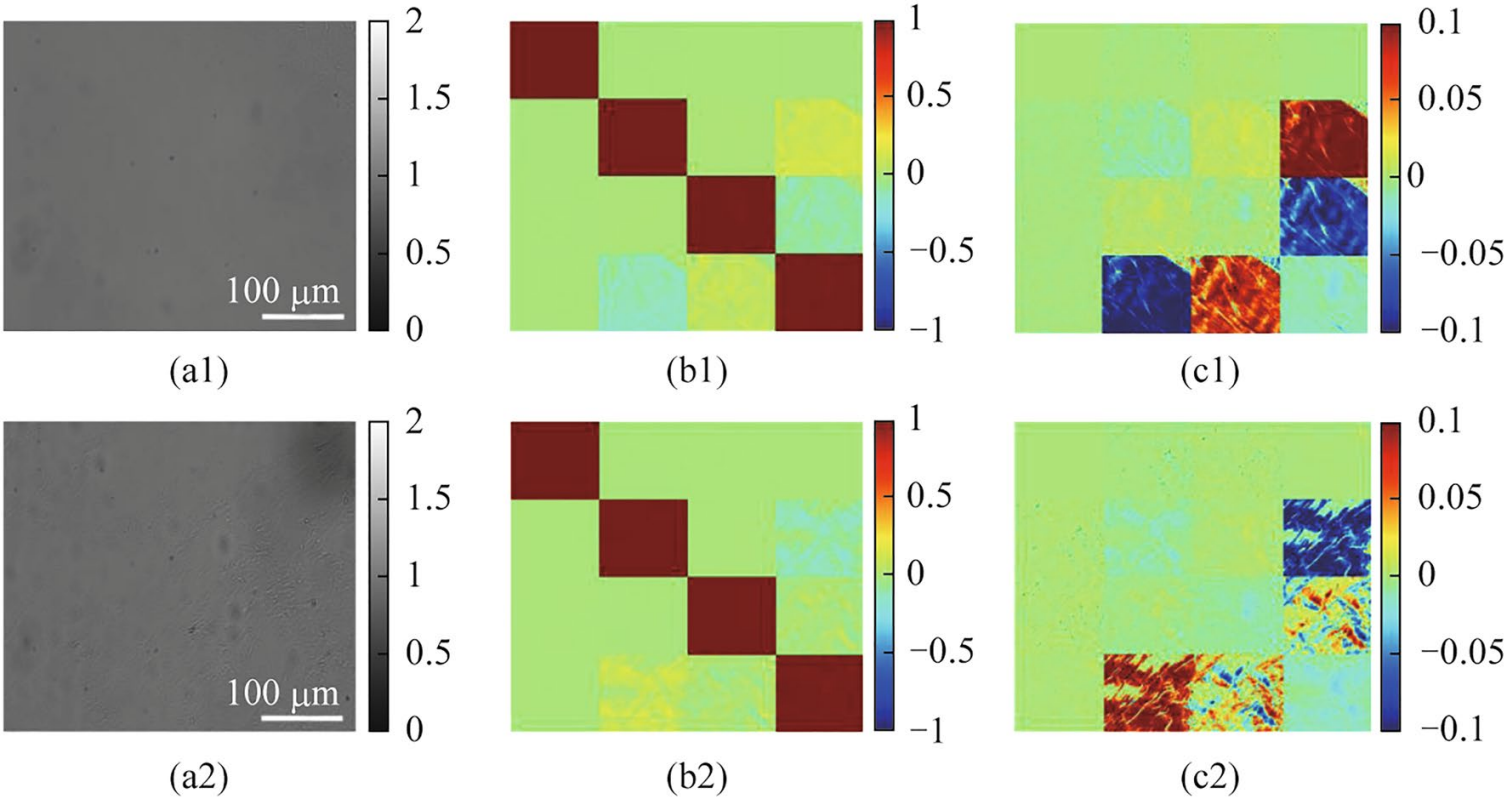
**a1**, **a2** Light intensity images, **b1**, **b2** MM images, and **c1**, **c2** MM (minus unit matrix) images for normal (the top row) and model groups (the bottom row)

Based on the MM microscopy, the MM images of the normal and injured model Achilles tendon mentioned in Section 2.1 are shown in Fig. 3.

In the intensity images of both normal and injured model Achilles tendon samples, it is hard to distinguish between the fibrous region and the air region, let alone the difference between normal samples and injured model samples. By contrast, we can easily notice the difference in multiple MM elements for normal and injured tendons. Therefore, the analysis of the Mueller matrix and its derived parameters can provide a new idea for tendon injury assessment. From Fig. 3b1, b2, c1, c2, it can be noticed that the right and bottom edges of the MM show clear image features, implying a strong anisotropy of the tendon. To clarify the physical interpretation of the overall high-dimensional characteristics of the Mueller matrix elements, the PBPs of MM need to be extracted. In previous studies of anisotropic biological samples similar to the Achilles tendon [15, 30, 31], the change of phase retardance is a major phenomenon, so the relevant parameters, such as *δ*, *r*_*L*_, need to be considered. More than that, the Achilles tendon samples show apparent diattenuation which is also closely related to the tendon status, so the diattenuation parameters including *D*_*L*_ and *D* should be analyzed. Furthermore, the tendon tissue has a typical fibrous structure, and its orientation distribution is a key microscopic feature, so the angle indicators extracted from polarization measurements, such as *α*_*r*_ and *θ* should be noted. All these selected PBPs will be comprehensively evaluated to extract key polarization differences that distinguish different statuses of tendons.

**Fig. 4:**
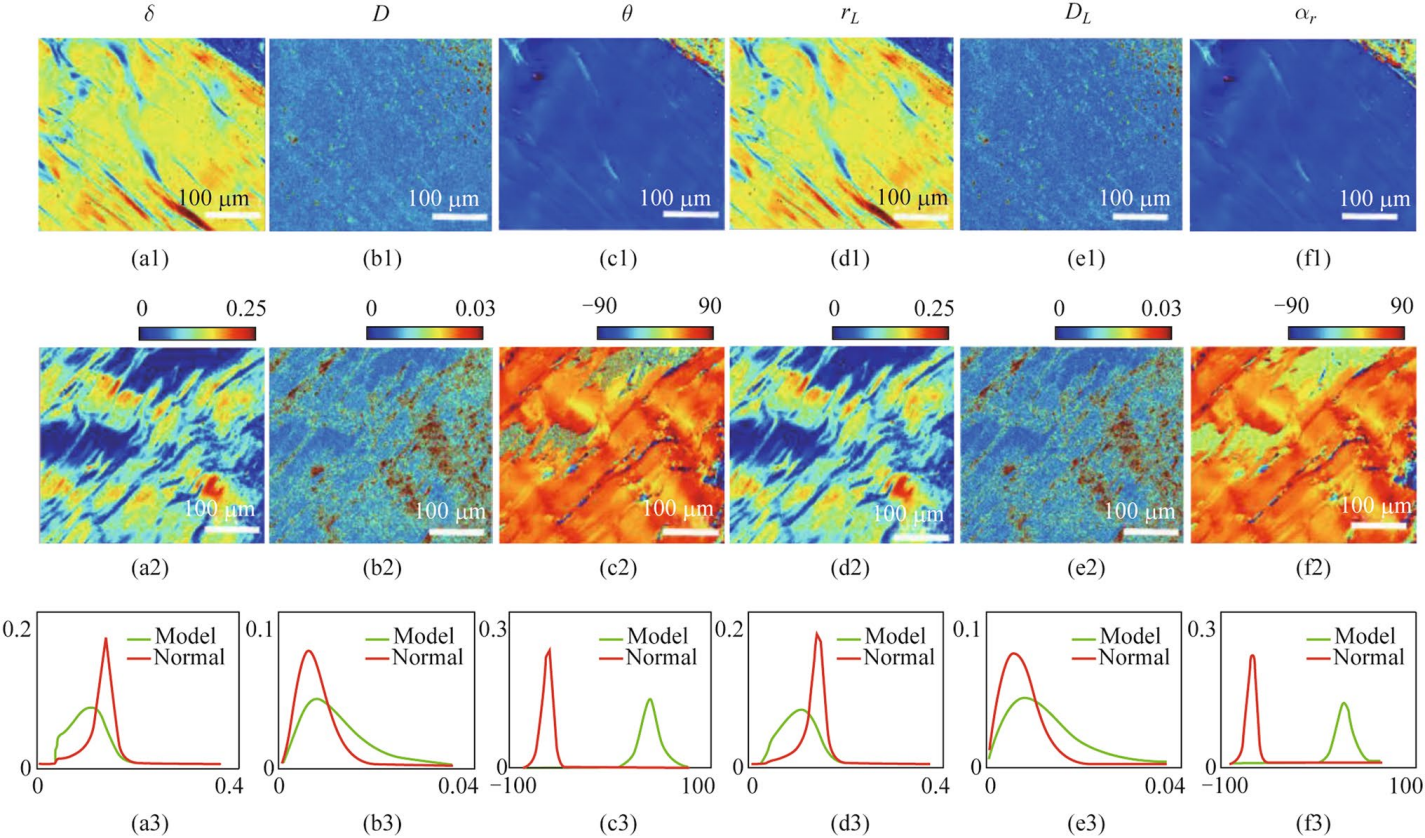
**a1**–**f1** Images of a selected normal group sample of $\delta$, $D$, $\theta$, $r_{L}$, $D_{L}$, and $\alpha_{r}$. **a2**–**f2** Images of a selected model group sample of $\delta$, $D$, $\theta$, $r_{L}$, $D_{L}$, and $\alpha_{r}$. **a3**–**f3** FDHs of the selected normal group sample and model group sample of $\delta$, $D$, $\theta$, $r_{L}$, $D_{L}$, and $\alpha_{r}$

Figure 4 presents six PBPs and their corresponding FDHs of normal and injured tendon samples. We can see a significant difference in PBPs between the two groups of non-stained Achilles tendon samples, confirming that polarization imaging has the advantage of discrimination significantly higher than that of non-polarized images. By observing the retardance parameters *δ* and *r*_*L*_, the values of healthy tendons are significantly higher than those of injury samples, and the distribution is more concentrated. Based on our previous studies, the retardance parameters with higher values tend to originate from dense fibers, which corresponds to the above-mentioned phenomena in Fig. 4a3 and d3. Next, the diattenuation parameters *D* and *D*_*L*_ of both two groups are sparsely distributed, whose value distribution in images cannot correspond strictly with the phase retardance images. And from Fig. 4b3 and e3, the diattenuation from the injured model is a little larger than that of the normal group. Then, for the angular parameters *θ* and *α*_*r*_, the average angle is closely affected by the sample position, so we mainly examine the statistical distribution characteristics of angle indicators. Intuitively, the healthy tendon is more uniformly oriented, and the injured model sample is more dispersed.

**Fig. 5:**
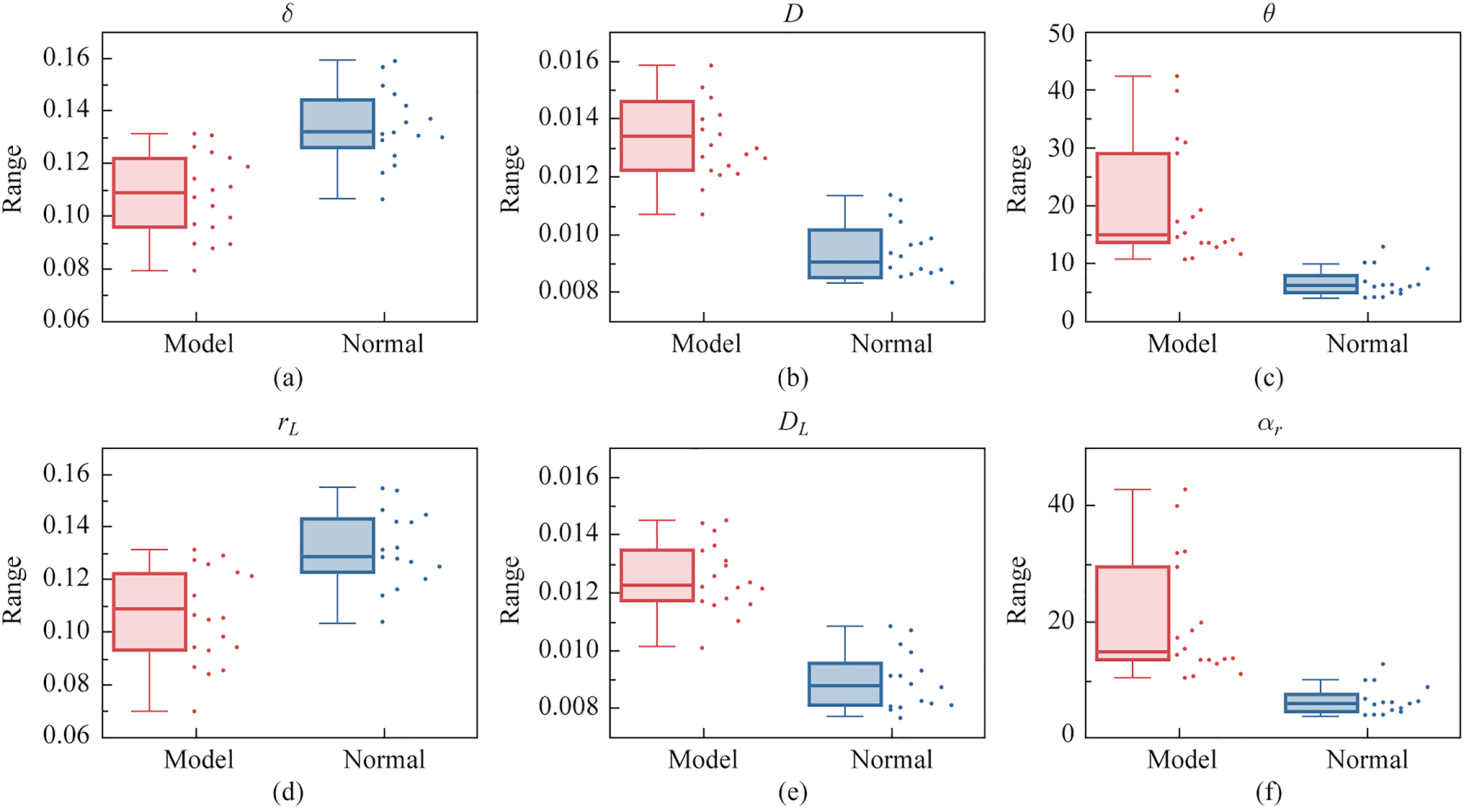
Statistical values of **a **$\delta$, **b **$D$, **c **$\theta$, **d **$r_{L}$, **e **$D_{L}$, and **f **$\alpha_{r}$ for the normal group and model group

Furthermore, the statistical parameters of the PBPs were calculated for all the samples within both groups, thereby illustrating the statistical difference that exists between the two categories of samples. For $\delta$, $D$, $r_{L}$, and $D_{L}$, their means were calculated, and for $\theta$, $\alpha_{r}$, their standard deviations were calculated. As shown in Fig. 5, there are significant differences between the two groups of samples on the box plots of all six polarization parameters. From Fig. 5, the injured tendon samples show smaller phase retardance values, larger diattenuation values, and wider fiber angle distributions. These statistical results confirm the feasibility of the three types of PBPs applied to diagnose whether there is tendon injury or not.

### Evaluation of Achilles tendon recovery based on PBPs

Based on the experiments in Section 2.1, we selected 10, 12, 13, and 15 regions of interest (ROI) in the images from low, medium, high, and positive experimental groups, respectively. We calculated the *p*_n_ of six PBPs based on a total of 16 ROIs from the normal group sample and then obtained the corresponding evaluation indicator *S*_parameter_ for each experimental group.

**Fig. 6:**
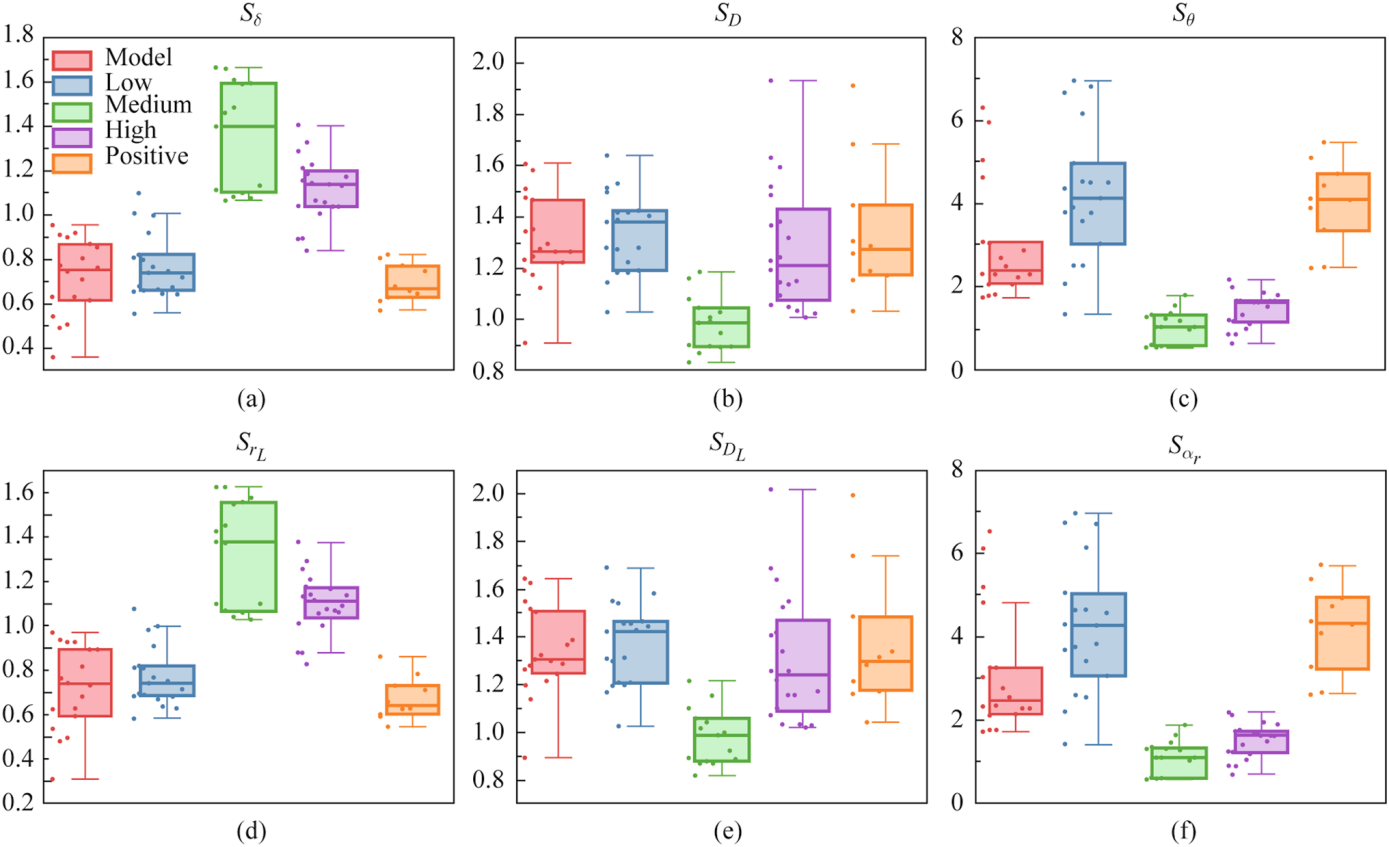
$S_{{{\text{parameter}}}}$ of the five experimental groups of **a **$\delta$, **b **$D$, **c **$\theta$, **d **$r_{L}$, **e **$D_{L}$, and **f **$\alpha_{r}$

For the retardance parameters, the values of healthy tendons are relatively large, so the recovery process shows an increase in this type of indicator. It can be reasonably inferred that the larger *δ* and *r*_*L*_ represent the better recovery effect. From Fig. 6a and d, the medium group shows the most prominent improvement compared to the model group, with the high group in second place, and the low and positive groups are not significantly different from the model group. It should be noted that the *S*_parameter_ values from both the medium groups are overall greater than 1, implying that from the perspective of phase retardance, the medium group can behave even better than the normal group.

For diattenuation parameters, taking normal samples as a reference, it can be deduced that effective repair therapy should be able to reduce the value of this type of parameter. From Fig. 6b and e, the overall diattenuation from the medium group is much smaller, and significantly better than that of the model group, followed by the high group, and there are no significant differences between the low, positive groups, and model group.

Finally, for the angular parameters, similarly by comparing injured model samples with normal samples, a better recovery effect should make the angular distribution of fibrous structure more consistent, meaning a smaller standard deviation of the angular parameters.

It can be seen from Fig. 6c and f that the standard deviations of angular parameters from the medium and high groups showed a very significant reduction in comparison with that of the model group, indicative of the improvement caused by the treatment. Whereas both the low and positive groups showed a widespread standard deviation, indicating the instability of the treatment effect.

### Monte Carlo simulation results

The Achilles tendon is an anisotropic tissue, and its internal fibrous structure causes cylinder scattering as well as birefringence, suitable for achieving contrast enhancement of microscopic features through polarization imaging. In Section 3.2, we observed significant differences reflected in three basic polarization parameters between the normal and the injured Achilles tendon samples.

Considering Monte Carlo simulation combined with a SCBM tissue model has shown its effectiveness in simulating and interpreting anisotropic tissues [11, 32], this study follows this way to explain polarization differences from the microscopic level, and then further describe the recovery mechanism of injured Achilles tendon under different treatment levels.

According to related studies [2, 33], our simulations need to consider the changes of fiber diameter, fiber orientation, and ECM during the tendon recovery process. The above pathological microscopic features correspond to the adjustment of the standard deviation of cylinder orientation distribution, the cylinder diameter, and the interstitial birefringence in the SCBM, respectively. From experiments, it can be seen that the standard deviation of the polarization parameter corresponding to tissue orientation angle is decreased after treatment, which can be understood as the fiber orientation in the repaired Achilles tendon is more consistent, consistent with other studies [8, 9, 33]. However, the increased retardance and the decreased diattenuation after treatment still need to be further connected with the changes in physiologic structure.

The SCBMs for the Achilles tendon are set as follows [34], the key parameters of the normal Achilles tendon involve a radius of 0.1 μm, the scattering coefficient of 50 cm^−1^ for spherical scatterers, and a fiber radius of 0.11 μm and the scattering coefficient of 180 cm^−1^ for cylindrical scatterers. Additionally, the refractive indexes of spherical and cylindrical scatterers are 1.39 and 1.47, respectively. The birefringence Δ*n* is set as 0.0013, and the optical axis of birefringence is along the tendon fibers, that is, the *x*-axis, consistent with the experimental tissue samples.

**Fig. 7:**
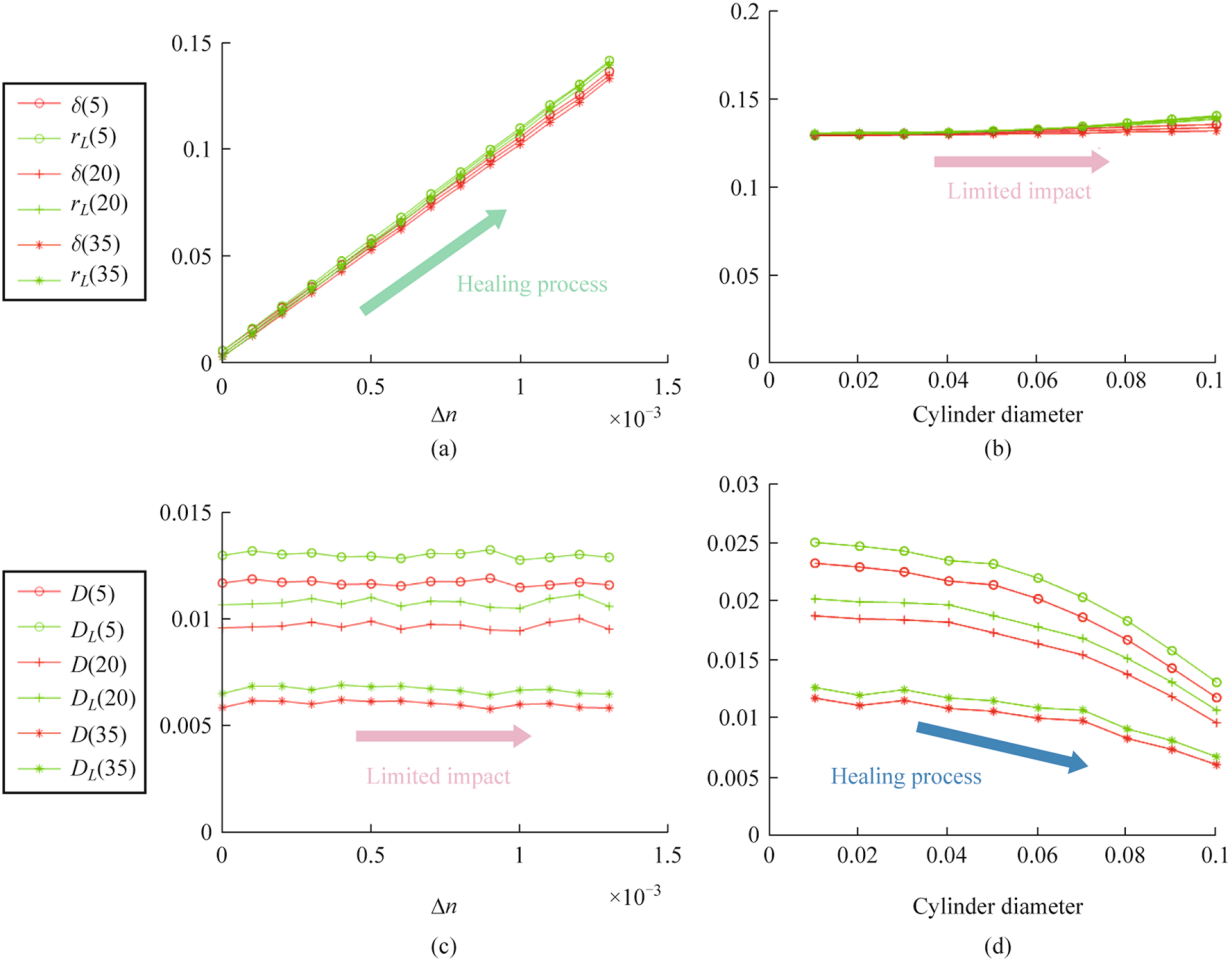
Monte Carlo simulation results. Values of retardance parameters with the change of **a** birefringence (Cylinder diameter is set to 0.1 μm) and **b** cylinder diameter (Birefringence is set to 0.013) when the standard deviation of cylinder orientation is set to 5, 20, and 35. Values of diattenuation parameters with the change of **c** birefringence (Cylinder diameter is set to 0.1 μm) and **d** cylinder diameter (Birefringence is set to 0.013) when the standard deviation of cylinder orientation is set to 5, 20, and 35

For tendon models with different fiber order degrees, Fig. 7a and b present the fluctuation of phase retardance with increasing birefringence and cylinder diameter, respectively. Apparently, the retardance parameter increases linearly with birefringence, while the increase in cylinder diameter and the standard deviation of cylinder orientation has only a limited impact. According to the reference [3], there is a quantitative relationship between collagen birefringence and its amount in tissues. Therefore, the increased phase retardance after treatment may be due to an enhanced birefringence, implying the increased collagen content.

In the above study, experimental results show a better consistency of fiber orientation after tendon repair treatment, corresponding to a decreased standard deviation of cylindrical scatterer orientation in the simulations. However, if we investigate the variation of the diattenuation parameter with cylinder orientation distribution from Fig. 7c and d, it can be seen that more ordered cylindrical scatterers lead to the increased diattenuation, which does not agree with experiments. At this point, we need to consider two other possible mechanisms in the tendon repair process, namely the increase of collagen fiber diameter and the enhanced birefringence due to the increased collagen content. Based on different standard deviation settings of collagen fiber orientation, Fig. 7c and d present the changes of diattenuation with increasing birefringence and cylinder diameter, respectively. It can be seen that the diattenuation decreases with increasing cylinder diameter, while the increase in birefringence has essentially no effect. Therefore, a reasonable explanation is that for the injured tendon repaired with treatment, the fiber orientation tends to be more orderly, and meantime the fiber diameter is increased. If the latter is a major factor, the overall diattenuation still shows a downward trend.

In summary, based on the compound effects of three microscopic factors, Monte Carlo simulations provide an explanation mechanism for the changes of three key polarization parameters in the recovery process of Achilles tendon injury, including the increase of fiber diameter, collagen content in ECM, and more ordered fiber orientation.

### Evaluation of Achilles tendon recovery based on PFP

The above-mentioned Fig. 2 has demonstrated the schematic for extracting PFP based on distribution differences from the Multidimensional PBP index system. In this section, to obtain the best linear weighting coefficients, we selected a total of 15 rotation-invariant PBPs. Considering the random placement of experimental samples, the exclusion of rotation-variant PBPs, such as *θ* and *α*_*r*_, can effectively avoid the interference of sample placement on anisotropic-related analysis. We establish a data set of all pixels 16 ROIs of the 4 normal samples as $C_{1}$, and another data set of all pixels of 22 ROIs of the 4 injured model samples as $C_{2}$.

We obtained the PBPs of the two sample groups by MM microscopy at 20 × magnification and divided all pixels of the two types of samples into two sets. By maximizing the distribution difference between two sets after calculating the PFP, the PFP based on linearly weighted PBPs is obtained. Moreover, the PFP can evaluate their discriminative capability according to the weighting coefficients of each PBP. Based on our previous polarization studies of fibrous tissue samples, we selected four rotation-invariant PBPs including *δ*, *D*, *r*_*L*_, and *D*_*L*_ for a comparative analysis of Achilles tendon samples. *θ* and *α*_*r*_ could only be used in the comparison between samples, however, these rotation-variant PBPs are not suitable for pixel-level information extraction. The distribution differences of PFP and PBPs are listed in Table 3, from which we can find that the PFP shows the largest discrimination between normal tendon and injured model samples, which is consistent with our prediction. So the extracted PFP, as a specific identification parameter, can be a diagnostic criterion for tendon injury.

**Table 3:** Distribution differences *F*(*W*) of PFPs and PBPs

Parameter name	$F$(*W*)
PFP	0.639
*δ*	0.340
${r}_{L}$	0.342
*D*	0.166
${D}_{L}$	0.176

**Fig. 8:**
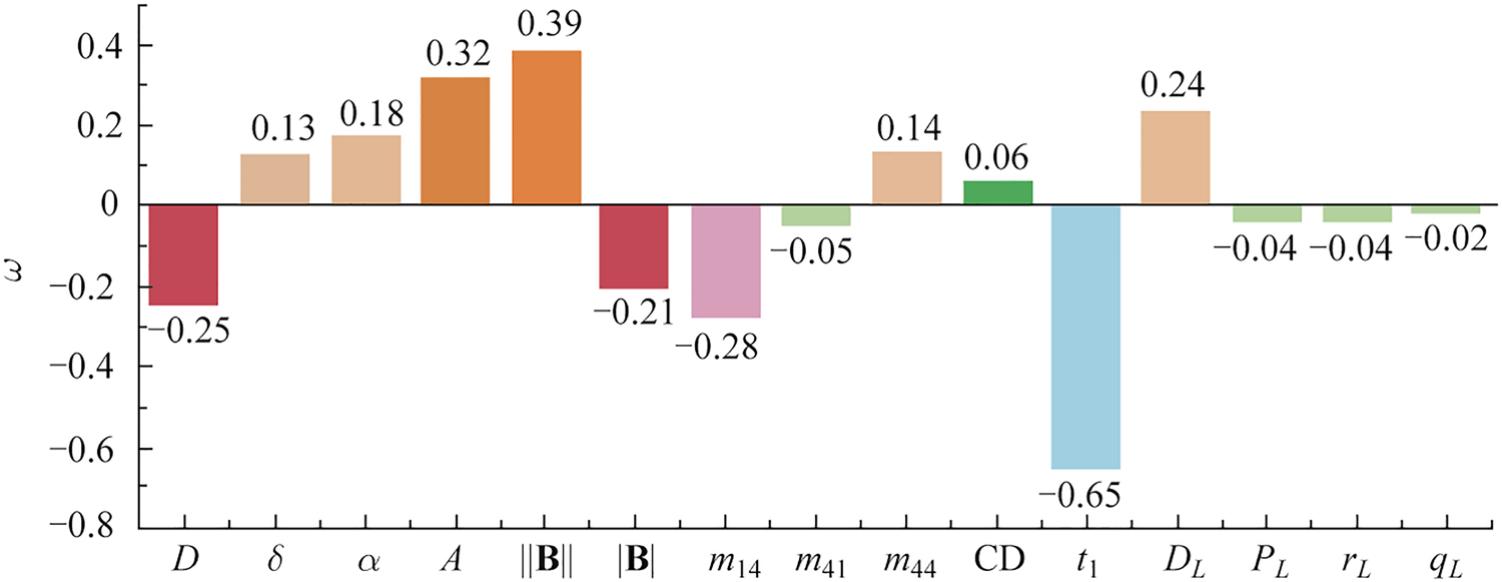
Weighting coefficient *ω* of PBPs

We further analyze the weighting coefficients of each PBP, as shown in Fig. 8, among the influential factors, *t*_1_ (− 0.65), || (0.39), $\| \mathbf{B}\|$ (− 0.21), and *A* (0.32) are associated with the overall anisotropy; *D* (− 0.25), *D*_*L*_ (0.24) and *m*_14_ (− 0.28) correspond to diattenuation; and *δ* (0.13) can describe linear retardance. Meanwhile, the differences in the weights of some PBP indicators should be noted and may provide reference bases for subsequent analysis and data mining. Concretely, the asymmetric contribution between MM elements, for example, *m*_14_ (− 0.28) and *m*_41_ (− 0.05) should be noted. Both *D*_*L*_ (0.24) and *P*_*L*_ (− 0.04) correspond to symmetric MM elements but have different weighting coefficients for distinguishing the tendon status.

**Fig. 9:**
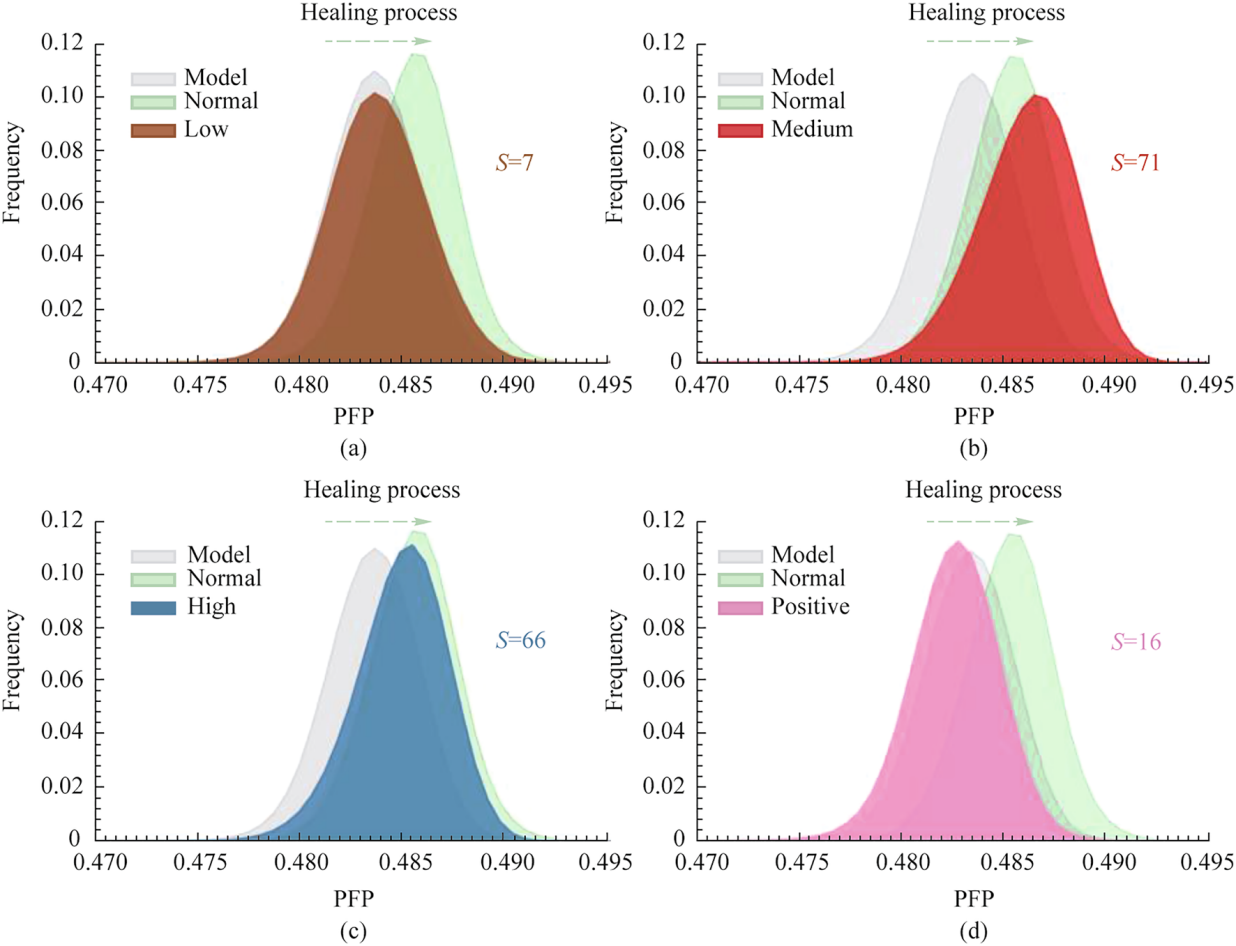
FDHs of **a** low, **b** medium, **c** high, and **d** positive group comparing with model and normal group in PFP

Further, based on the FDH curves for each sample group, PFP can be used to evaluate the effects of different treatment regimens. As shown in each subgraph of Fig. 9, the FDHs of four treatment groups are drawn and compared with the FDHs of the injured model and normal group. It can be seen that an effective tendon repair should shift the PFP distribution curve to the right close to the healthy group. The results of the high and medium treatment groups are closer to those of the normal group, and the low and positive groups are similar to those of the model group, confirming the better treatment effect of the tendon repair schemes in the medium and high treatment groups. By a comprehensive evaluation based on the average value and distribution width of the specific polarization parameter, the medium group was the best treated, the high group was the second best, and the low and positive groups had poor efficacy.

**Fig. 10:**
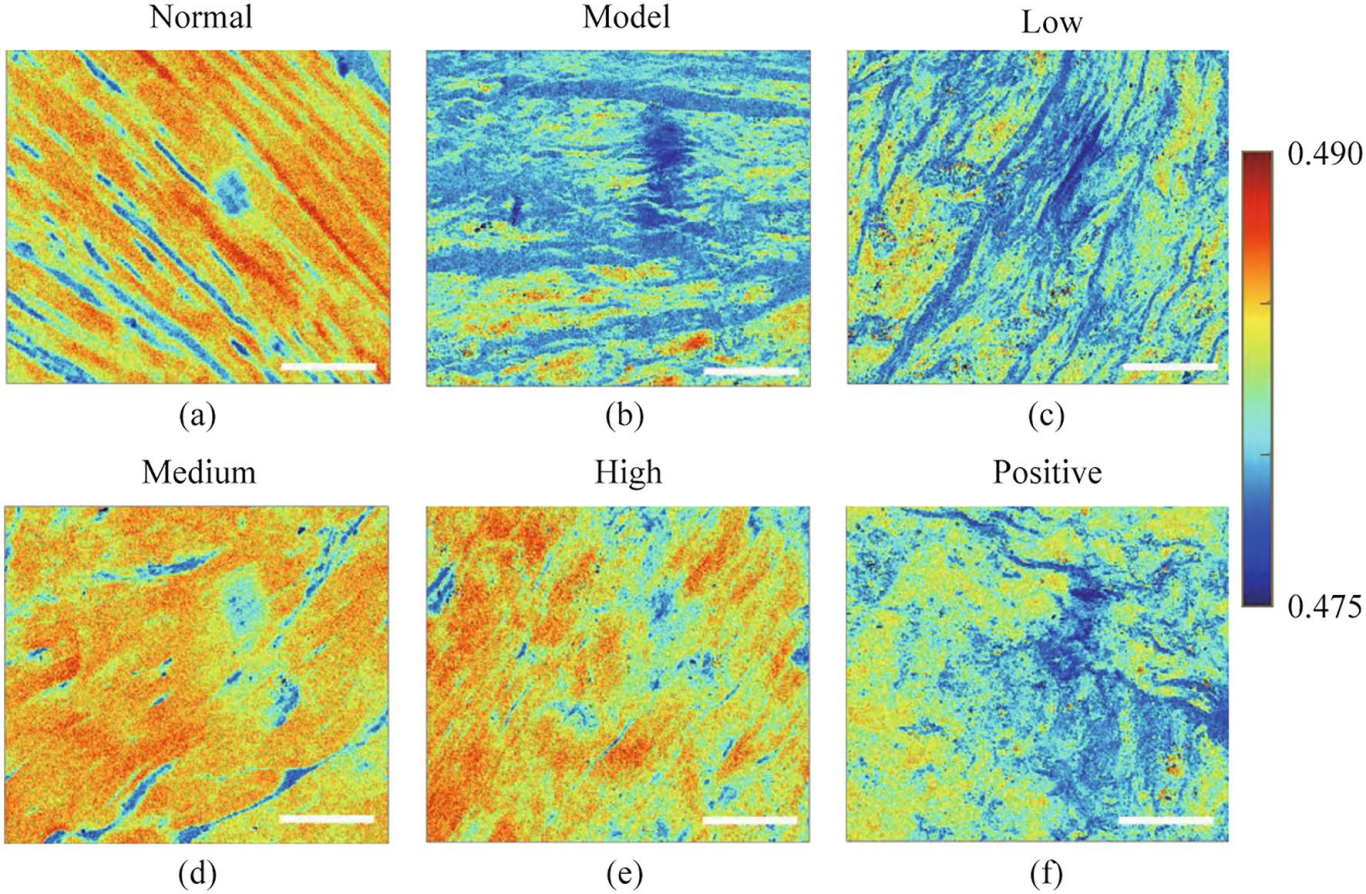
PFP as a result of polarization staining **a** normal group, **b** model group, **c** low group, **d** medium group, **e** high group, and **f** positive group. The white scale bar is 100 μm

The above research shows that those high-value areas in a PFP image can indicate healthier tendon parts. We briefly show the PFP results of each group as polarization staining in Fig. 10, the orange-red color corresponds to the high-value regions, the green takes the second place, and blue indicates the lowest value. Accordingly, PFP images provide a new way to label tendon status without staining, where the orange-red parts indicate the distribution of healthy tendons, the green parts suggest injured tendons, and the blue corresponds to the air region. From Fig. 10, we can intuitively understand the state distribution of tendon samples, where higher quality tendons are more widely distributed in images of normal, medium, and high groups and show better consistency. On the contrary, the high-quality tendons are rarely seen in images of the model, positive control, and low groups. Figure 10 demonstrates the potential of PFP as a virtual staining parameter applied in label-free tendon diagnosis and treatment evaluation.

## Conclusions

This study utilized Mueller matrix imaging technology to perform full polarization microscopic analysis and feature extraction on various Achilles tendon injured samples. The experiments observed three key polarization basic parameters closely related to Achilles tendon injury, that is, phase retardance, diattenuation, and fiber orientation angle. Specifically, healthy Achilles tendons exhibit greater phase retardance, smaller diattenuation, and smaller standard deviation of orientation angle distribution.

Based on these findings, this study provides an in-depth evaluation of Achilles tendon recovery in different treatment-level groups. Then with the help of Monte Carlo simulations, the relevance between experimental phenomena and microscopic changes in injured Achilles tendons was explained. Further research extracted a specific indicator PFP for Achilles tendon injury from the key basic polarization parameters mentioned above, which has been confirmed as a more effective discriminator suitable for recovery evaluation for different treatment groups. Moreover, a label-free polarization staining using PFP offered an intuitive image recognition for Achilles tendon injury and recovery, implying its potential in clinical applications.

## Data Availability

The data that support the findings of this study are available from the corresponding author, upon reasonable request.
